# Automated inference of Boolean models from molecular interaction maps using CaSQ

**DOI:** 10.1093/bioinformatics/btaa484

**Published:** 2020-05-13

**Authors:** Sara Sadat Aghamiri, Vidisha Singh, Aurélien Naldi, Tomáš Helikar, Sylvain Soliman, Anna Niarakis

**Affiliations:** GenHotel, Département de Biologie, Univ. èvry, Université Paris-Saclay, Genopole, èvry 91025, France; GenHotel, Département de Biologie, Univ. èvry, Université Paris-Saclay, Genopole, èvry 91025, France; Département de Biologie, Institut de Biologie de l'Ecole Normale Supérieure (IBENS), ècole Normale Supérieure, CNRS, INSERM, Université PSL, Paris 75005, France; Department of Biochemistry, University of Nebraska-Lincoln, Lincoln, NE 68588, USA; Lifeware Group, Inria Saclay-île de France, Palaiseau 91120, France; GenHotel, Département de Biologie, Univ. èvry, Université Paris-Saclay, Genopole, èvry 91025, France

## Abstract

**Motivation:**

Molecular interaction maps have emerged as a meaningful way of representing biological mechanisms in a comprehensive and systematic manner. However, their static nature provides limited insights to the emerging behaviour of the described biological system under different conditions. Computational modelling provides the means to study dynamic properties through *in silico* simulations and perturbations. We aim to bridge the gap between static and dynamic representations of biological systems with CaSQ, a software tool that infers Boolean rules based on the topology and semantics of molecular interaction maps built with CellDesigner.

**Results:**

We developed CaSQ by defining conversion rules and logical formulas for inferred Boolean models according to the topology and the annotations of the starting molecular interaction maps. We used CaSQ to produce executable files of existing molecular maps that differ in size, complexity and the use of Systems Biology Graphical Notation (SBGN) standards. We also compared, where possible, the manually built logical models corresponding to a molecular map to the ones inferred by CaSQ. The tool is able to process large and complex maps built with CellDesigner (either following SBGN standards or not) and produce Boolean models in a standard output format, Systems Biology Marked Up Language-qualitative (SBML-*qual*), that can be further analyzed using popular modelling tools. References, annotations and layout of the CellDesigner molecular map are retained in the obtained model, facilitating interoperability and model reusability.

**Availability and implementation:**

The present tool is available online: https://lifeware.inria.fr/∼soliman/post/casq/ and distributed as a Python package under the GNU GPLv3 license. The code can be accessed here: https://gitlab.inria.fr/soliman/casq.

**Supplementary information:**

Supplementary data are available at *Bioinformatics* online.

## 1 Introduction

### 1.1 Biological network representations and molecular interaction maps

Biological phenomena can be viewed in the form of interaction networks where components (genes, proteins) are represented as ‘nodes’ and the interactions between components are represented as ‘edges’. Network interactions can be directed or undirected, depending on the biological information available that allows the characterization of the interaction (inhibition or activation) and also the source and the target node. Representing the complexity of biological regulatory systems using networks enables the analysis of their topology, identifying distinct clusters that may correspond to specific biological processes (‘modules’) and nodes with a high degree of connectivity (‘hubs’), exercising a significant influence on the propagation of biological information (i.e. signal, regulation) (Barabási and Oltvai, 2004; Ideker and Nussinov, 2017; Zhang *et al.*, 2014).

The Systems Biology Graphical Notation (SBGN) scheme uses three different languages for network representation (Le Novère, 2015). First, the activity flow (AF) diagram that is an interaction network, which includes influence direction and mode of regulation, such as activation and inhibition. Second, the entity-relationship (ER) representation that includes mechanistic details, the direction of influences but no sequential information and third, the process description diagram (PD) which is the most detailed of all, including details of the direction of influences, mechanism of action and the order of events. The SBGN-PD notation scheme is based on ideas first introduced to the field by Hiroaki Kitano and co-workers (2003).

Molecular interaction maps can be used to describe biological mechanisms concisely and effectively. Various molecular maps describing different biological processes (Caron *et al.*, 2010; Fujita *et al.*, 2014; Grieco *et al.*, 2013; Jagannadham *et al.*, 2016; Kuperstein *et al.*, 2015; Mazein *et al.*, 2018; Niarakis *et al.*, 2014; Ogishima *et al.*, 2016; Singh *et al.*, 2018; Tripathi *et al.*, 2015; Singh *et al*., 2020) have been published, and initiatives have emerged, such as the Disease Maps Project (http://disease-maps.org), demonstrating the utility and need of this type of representation of biological knowledge (Mazein *et al.*, 2018; Ostaszewski *et al.*, 2019). Molecular interaction maps can serve as a stand-alone knowledge base, or they can be used as a scaffold for building computational models. Based on information mining, human curation and expert advice, these maps summarize current knowledge about biological pathways in a process description representation, while accounting for as many mechanistic details as possible. They provide a comprehensive template for visualization and analysis of omics datasets, and can also be analyzed in terms of the underlying network structure. However, their static nature cannot account for the coordination of multiple biological processes, or how the regulation of several nodes due to the presence or absence of certain factors can alter the functional outcome (i.e. activation of a particular pathway following the repression of a given factor). These regulations that fine-tune the molecular interactions are of great importance as dysregulation or disruption can lead to disease (Cho *et al.*, 2012; Furlong, 2013).

### 1.2 Boolean models for dynamical studies

Systems Biology approaches and especially computational modelling can be used to provide an executable, dynamic network that can reveal hidden properties and account for emerging system-level behaviours through *in silico* simulations and perturbations (Azeloglu and Iyengar, 2015; Helikar *et al.*, 2008). Each interaction is described using mathematical formalism and the obtained machine-readable model can be used to test novel hypotheses and predict new features of the system of interest. Boolean models are well suited for addressing the lack of kinetic data and handling the large size of the biological pathways described in molecular interaction maps. These models are parameter-free; nevertheless, their simplistic nature can provide a powerful tool for dynamic analysis (Abou-Jaoudé *et al.*, 2016; Furlong, 2013). In Boolean formalism, the simplest form of logical models, nodes represent regulatory components (proteins, enzymes, complexes, transcription factors, genes, to name a few) and arcs represent their interactions. Each regulatory component is associated with a Boolean variable (taking the values 0 or 1) denoting either its qualitative concentration (0 for absent or 1 for present) or its level of activity (0 for inactive or 1 for active). The future state of each node depends on the state of its upstream regulators and is defined by a Boolean function. The function is expressed in the form of a rule using the logical operators AND, OR and NOT. The updating of the rules can be in a synchronous, deterministic mode where all nodes are updated at the same time (Glass and Kauffman, 1973; Kauffman, 1969) or in an asynchronous mode, where only one node can be updated every time (Thomas, 1973, 1978; Thomas *et al.*, 1976).

### 1.3 Bridging the gap between static and dynamic representations

The construction of a molecular interaction map and a dynamic model are two tasks that can serve different purposes and are usually performed independently. On the one hand, it is a question of creating a knowledge base in the form of a comprehensive molecular map, and on the other of defining the underlying mechanism that links the system components and captures its dynamic behaviour. Nevertheless, these two constructs share much information, including the mode of influence (e.g. activation or inhibition) and the topology of the network. Molecular maps can be built using a structured diagram editor for drawing gene-regulatory and biochemical networks, such as CellDesigner (Funahashi *et al.*, 2003). Networks in CellDesigner are drawn as process description diagrams (PD) and are stored using the Systems Biology Markup Language (SBML), a standard for representing models of biochemical and gene-regulatory networks (Hucka *et al.*, 2003).

The idea of obtaining executable models from a network topology is not new. In the study by Büchel *et al.* (2013), researchers proposed a pipeline for the automatic generation of models using KEGG pathways as a resource. They succeed in producing SBML and Systems Biology Marked Up Language-qualitative (SBML-*qual*) files but these constructs can be seen as model scaffolds as they require further parameterization to become executable. In Mendoza and Xenarios (2006), a Standardized QUAlitative Dynamical system (SQUAD) is obtained directly from an input network that is already a regulatory network and not a molecular interaction map. Furthermore, the aim is to obtain a continuous system corresponding to it, implying a small-scale network (about 20–30 nodes). Regarding Biolayout, now Graphia (Livigni *et al.*, 2018), researchers use the modified Edinburgh Pathway Notation scheme (mEPN) to create SBML-like maps that they interpret directly as Petri nets. This approach imposes that all ‘logics’ are conjunctive. There is no direct negation, no disjunction, whereas the only firing rule in a Petri net is that all input places should be filled in order for the reaction to fire. However, molecular maps contain much more precise information (e.g. inhibitions) that cannot be expressed directly within this framework. Moreover, Petri nets are by nature quantitative, requiring several tokens to be assigned to each place, and having the consumption of some tokens by each rule. The rxncon language (Romers and Krantz, 2017) also tackles the idea that there are standard features between maps as knowledge-bases and executable Boolean models. However, their approach is quite different from ours in that they bridge this gap through an intermediate language based on Boolean bipartite graphs. One of the most important consequences is that the logical rules (contingencies in rxncon) are already part of the input (the map being, in a way, already a model). Finally, the http://pd2af.org/ initiative (Vogt *et al.*, 2013) proposes to translate an SBGN-PD graph, similar to a CellDesigner map, into an SBGN-AF graph, similar to the structure of a Boolean model, but does not go further as to propose an executable model. We will detail in the discussion some specific rules for which we have made similar or opposite choices concerning the graph transformation. However, one should note that our method adds the layer of inferring logical rules for the obtained model based on the original topology and annotations, making possible immediate simulations and analyses using the corresponding tools [e.g. GINSim (Chaouiya *et al.*, 2012) and Cell Collective (Helikar *et al.*, 2012)].

In this work, we present CaSQ (CellDesigner as SBML-*qual*), a tool for automated inference of large-scale, parameter-free Boolean models, from molecular interaction maps with preliminary logic rules based on network topology and semantics. CaSQ is, to the best of our knowledge, the first tool that produces executable molecular networks of hundreds of nodes (at least up to eight hundred), in the SBML-*qual* format that can be further simulated and analyzed using popular modelling tools.

## 2 Materials and methods

### 2.1 CaSQ

**Fig. 1.**
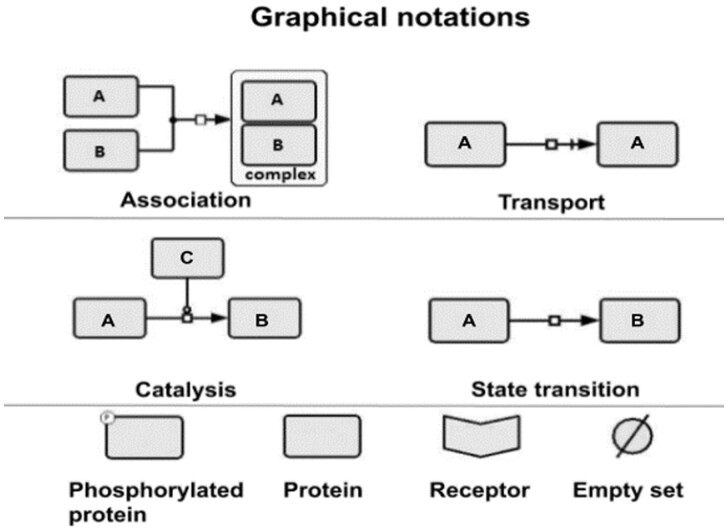
The repertoire of CellDesigner graphical notation schemes used to illustrate CaSQ’s rules. For CaSQ’s conversion rules, we use the notation schemes for association, transport, catalysis, state transition and also the glyphs for receptor, protein, modified protein (here, we show phosphorylation as an example) and the empty set. The empty set can account for degradation or in SBGN-PD terms, can represent the creation (respectively, the disappearance) of an entity from an unspecified source (resp. sink) that we do not need or wish to explicit

CaSQ is a tool that can convert a molecular interaction map built with CellDesigner (Funahashi *et al.*, 2003) to an executable Boolean model. The tool is developed in Python and uses as source the xml file produced by CellDesigner (SBML plus CellDesigner-specific annotations) to infer preliminary Boolean rules based solely on network topology and semantic annotations (e.g. certain arcs are noted as catalysis, inhibition, etc.). The aim is to convert a Process Description (PD) representation, i.e. a reaction model, into a complete logical model. The resulting structure is closer to an AF diagram, though not in a strict SBGN-PD to SBGN-AF notion. Moreover, logical rules that make the model executable are also obtained. For illustrating the rules of the conversion, we use the repertoire of notation schemes in CellDesigner (Fig. 1).

**Fig. 2.**
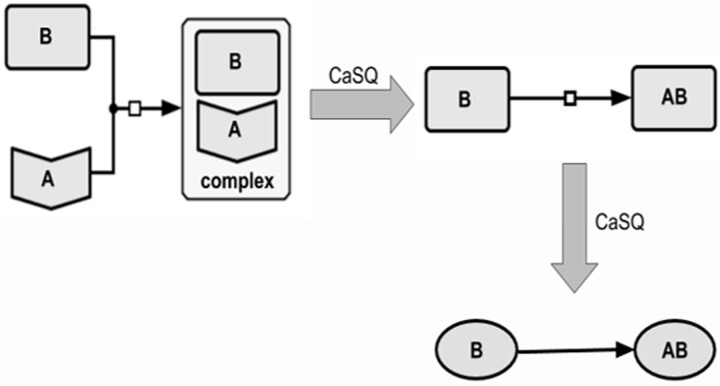
Illustration of the 1st rule. If two species of the map are only reactants in a heterodimer association, and if one of the reactants is annotated as a receptor, then the receptor is deleted from the map (its annotations are added to the product of the reaction)

**Fig. 3.**
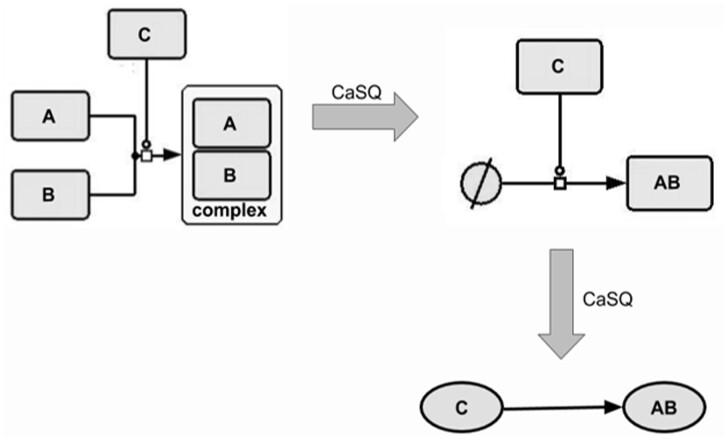
Illustration of the 2nd rule. Compression of the complex formation, where none of the reactants is denoted as a receptor, and both reactants do not participate in any other reaction. As a result, both reactants are removed and modifiers are rewired to have the complex as a product

**Fig. 4.**
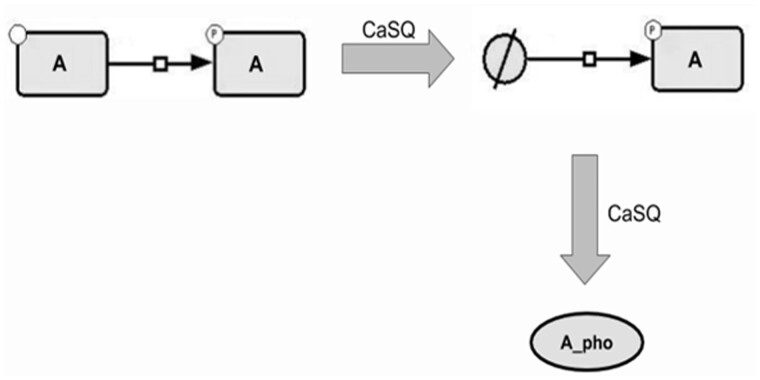
Illustration of the 3rd rule. Removing inactive forms that do not participate in other reactions

**Fig. 5.**
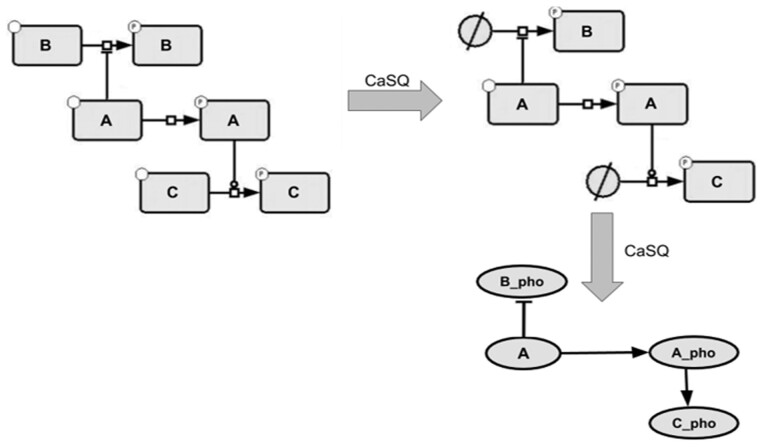
Combination of rules 2 and 3. CaSQ retains components that contribute further to the propagation of the signal

The conversion of the graph to an executable model is a four-step process:

**Step 1:** First, the map is reduced through a pass of graph-rewriting rules. These rules are executed in order and in a single pass, so the rewriting is terminating and confluent. The reasoning behind this reduction is that a single qualitative species of the logical model often represents by its state (active/inactive) several species of the original map. Therefore, those species might need to be merged into a single component or some inactive forms to be completely discarded to avoid redundancy in the logical model. The rules are the following:

**Rule 1:** If two species of the map are only reactants in a single reaction, i.e. do not take part in any other reaction, if that reaction is annotated as heterodimer association, and if one of the reactants is annotated as a receptor, then the receptor is deleted from the map (its annotations are added to the product of the reaction) (Fig. 2);

**Rule 2:** If two species of the map take part in a reaction annotated as heterodimer association, **if none of them** are annotated as receptor, and **if both** do not take active part (i.e. reactant or modifier) in any other reaction, then both are merged into the complex, product of the reaction (their annotations are added to the product, and the reactions that had them as product are rewired to have the complex as product) (Fig. 3);

**Rule 3:** If one species only appears in a single reaction, if it appears there as a reactant if that reaction has a single product, and if both the reactant and the product have the same name, then the reactant is deleted (its annotations are merged into those of the product) (Fig. 4);

Rules 2 and 3 can be combined resulting in greater graph compression, as illustrated in Figure 5.

**Fig. 6.**
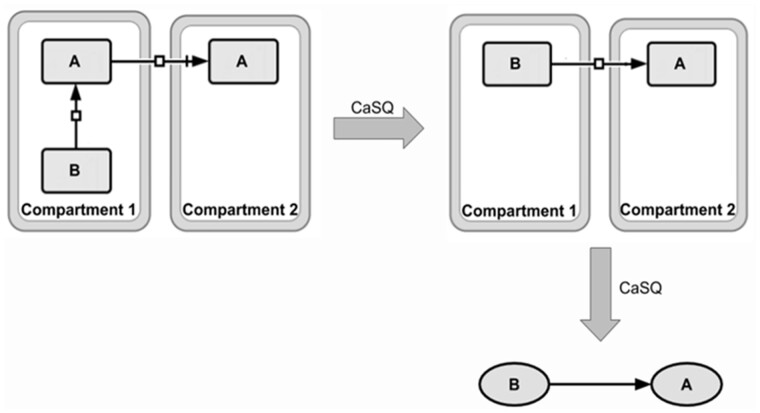
Combination of the 2nd and the 4th rule. Components that are translocated across other compartments (e.g. transcription factors) are merged in one component that inherits all influences, provided that the original component does not participate in another reaction/regulation

**Rule 4:** If one species only appears as a reactant in a single reaction (but maybe appearing as product in another reaction) that has a single product and is annotated as transport, and if both the reactant and the product have the same name, then the reactant is merged into the product (its annotations are merged into those of the product, and the reactions producing it are rewired to the product) (Fig. 6).

The rationale of using the name to identify the same components in different states (gene, RNA, protein, transported/phosphorylated/methylated protein, etc.) is that we need to identify when species can be merged/discarded, to keep only what contributes further to signal propagation. However, relying on the *active* annotation (dotted circle) in CellDesigner maps proved to be insufficient: not all map curators use this notation, and it is not SBGN compliant.

**Step 2:** The topology of the model is then computed as a simple form of PD to AF conversion, with one qualitative species corresponding to each species in the reduced map obtained from Step 1. This species inherits the original map layout, using SBML3 Layout package, and MIRIAM annotations (e.g. PubMed IDs as bqbiol: isDescribedBy). The annotations have been associated with each regulated component rather than each regulation, mostly because tools supporting the latter are quite rare. All reactants and modifiers of a reaction exert a positive influence on all the products of that reaction, whereas all inhibitors exert a negative influence. Compared to the formal abstraction of influence graphs from reaction graphs (Rizk *et al.*, 2011), note that, the mutual inhibition between reactants is purposely ignored as in Step 1 we already condense active and inactive forms of the same species.

**Step 3:** The logical rules of the model are computed. For each species, its logical rule is defined as the (i) disjunction (OR), for all reactions producing it, of (ii) the disjunction (OR) for all positive modifiers of a reaction being on and (iii) the conjunction (AND) of all products of that reaction being activated and all inhibitors being inactive. Therefore, a target is on if one of the reactions producing it is on, a reaction is on if all reactants are on, all inhibitors are off and one of the catalysts is on (Supplementary Fig. S1).

**Step 4:** Model refinement is performed through the optional removal of unconnected components. From our experience, keeping only the biggest connected component is what makes the most sense from a modelling perspective. However, it is possible to specify a ‘minimum size’ and keep all connected components above that size. Names of the qualitative species are also made more precise by adding the original type/modifications of the species (e.g. RNA, phosphorylated) and if there are still homonyms the original compartment is added too. More precisely, the name of the node in the model is, therefore, the name of the species in the map to which is added (separated by an underscore character ‘_’), its type as given in the map (RNA, Gene, etc.) unless that type is ‘PROTEIN’ and to which is added modifications given by the map (phosphorylation, methylation, etc.). If after that step, several species from the model are found to have the same name, the compartment is added too (once again, separated by an underscore) (Supplementary Tables S1 and S2).

CaSQ generates two output files; the proper logical model encoded in SBML-*qual*, a format that is compatible for further analysis with modelling tools such as GINsim (Chaouiya *et al.*, 2012) or Cell Collective (Helikar *et al.*, 2012), and a CSV file that contains information about the names, the logic formulae and the CellDesigner alias. The second file is mostly for automated treatment. The SBML-*qual* file can also be restricted to include only its biggest connected component (BCC), or only connected component above a given size threshold. This allows the modeller to obtain a more meaningful logical model even if the original map did contain several unconnected clusters corresponding to isolated pieces of information.

### 2.2 Molecular interaction maps and logic models

For testing the applicability of CaSQ, we used various molecular interaction maps that differ in size, complexity and use of SBGN notation, as shown in Table 1. Namely, we used one molecular interaction map comprising 125 nodes describing mast cell activation (Niarakis *et al.*, 2014), one map comprising 232 nodes for MAPK activation (Grieco *et al.*, 2013), one for cholecystokinin signaling with 530 nodes (Tripathi *et al.*, 2015) and finally two large-scale molecular maps, one for rheumatoid arthritis (RA)—the only SBGN-compliant—(Singh *et al.*, 2018, 2020) comprising 779 nodes, detailed annotations and references in the MIRIAM and text annotation section of the CellDesigner file (Funahashi *et al.*, 2003) (Supplementary Fig. S2) and the Alzheimer’s pathway map with 1361 nodes (Ogishima *et al.*, 2016). The mast cell activation and the MAPK maps were published along with their corresponding manually built logical models.

**Table 1.** Size (number of components) of the CaSQ-inferred model using the default and BCC options

Map name	Map size	SBGN use	CaSQ-inferred model			
			Size	Graph reduction (%)	BCC size	Graph reduction(%)
Mast cell^a^	125	No	80	36	73	42
MAPK^a^	232	No	182	21	181	22
Cholecystokinin	530	No	404	24	383	28
RA	779	Yes	431	45	391	50
Alzheimer’s	1361	No	1169	14	762	44

a The existence of a corresponding manually built logical model.

### 2.3 Model comparison

For evaluating the performance of the tool, we compared size and shared nodes between manually built models that corresponded to the interaction maps (for mast cell and MAPK), with the CaSQ-inferred Boolean models. While size reduction is not the primary goal of the tool, it remains a measure of comparison between the process description static diagram of the original map and the regulatory graph that the tool produces after the conversion rules. Conversion from a process description to an AF diagram implies a more compact network. The comparison allows us to check if such compression was achieved. We also performed simulations to see if the CaSQ-inferred models were able to reproduce known biological scenarios, and finally, we compared steady states, where feasible, between the inferred and the manually built models.

### 2.4 *In silico* simulations and calculation of stable states

For the simulations of the CaSQ-derived models, we used Cell Collective, a web-based, modelling platform for the collaborative construction, simulation and analyses of large-scale dynamic models (Helikar *et al.*, 2012). Models in Cell Collective can be created either *de novo* or they can be imported using the SBML-*qual* standard. Cell Collective SBML-*qual* import supports network layout, as well as model annotations. References stored in the MIRIAM section of the xml file of CellDesigner can be retrieved and visualized in the platform (Supplementary Fig. S3).

For the computation of stable states, we used GINsim (Chaouiya *et al.*, 2012), powerful software for constructing and analyzing logical models. GINsim can import SBML-*qual* files; however, it needs a pre-processing step to display the name and not the species IDs. Imported models retain their formulae, as well as the layout but are currently stripped from annotations during pre-processing.

## 3 Results

### 3.1 Graph reduction and model inference

We first tested the tool with different molecular maps of various sizes, complexities and use of standards to see if CaSQ was able to produce corresponding executable models. We performed the analysis with CaSQ first by default and then using the BCC option. While a model should be connected to be useful, a map can include unconnected parts as the objective of a map is to represent all current knowledge for the studied biological process and this knowledge is more likely to be fragmented. The purpose of using CaSQ with default and BCC options was also to evaluate the graph reduction capacities of the tool. The size was defined by the number of nodes included in the map (number of species in the CellDesigner files), and the number of components included in the published, manually built or CaSQ-inferred models.

CaSQ was able to handle small-, medium- and large-scale maps (ranging from 125 to 1361 nodes) with or without SBGN standards, and produce executable models smaller in size, offering a graph reduction of 21–45%. Using the BCC option that allows keeping the biggest connected component, the resulting models are slightly smaller. The size of the produced model—in terms of the number of components included—using BCC option is highly dependable on the connectivity of the initial map (Table 1).

### 3.2 CaSQ run time

The analysis was performed on a Dell working station with Windows 7, 64-bit Operating System, Installed memory (RAM): 64.0 GB and Processor: Intel (R) Xeon (R) CPU E5-1650 v4 @ 3.60 GHz 3.60 GHz. The run times of CaSQ for producing executable SBML-*qual* files with default options are 1.42 s for the mast cell activation map, 1.10 s for the MAPK map, 1.71 s for the Cholocystokinin, 2.29 s for the RA map and 5.24 s for the Alzheimer’s map.

### 3.3 CaSQ-inferred Boolean models versus manually built models

#### 3.3.1 Shared nodes

To evaluate the tool’s ability to produce preliminary Boolean rules, we compared the CaSQ-inferred models with the manually built models (MM) published with the respective maps. First, we compared the size and graph reduction percentage (Table 2). For the size, we compared the shared nodes between the two models. The automated comparison gives the number of identical node names while the manual comparison accounts for differences in node names that derive from the fact that the manually built models do not correspond 100% to the maps. A modeller may choose to merge two nodes (i.e. receptor–ligand), change the name of one node (i.e. use capitals or add underscores for a complex), entirely skip it or add a node that does not exist in the initial map, making it difficult to evaluate in a fully automated way the correspondence between the manually built and the CaSQ-derived models. Manual comparison by visual inspection after the automated comparison revealed many cases where the node names were slightly different but corresponded to the exact protein or gene (Supplementary Tables S1and S2). For example regarding the mast cell activation models, the manual model has RAS but the CaSQ model has H-RAS. Other cases concern grouping of instances, i.e. FYN in the manually built model corresponds to more instances in the CaSQ one, as the latter includes FYN with different modifications (phosphorylated, palmitoylated). For the MAPK model, an example is p53 in the manual model that corresponds to TP53 and TP53 phosphorylated in the CaSQ counterpart, or SMAD in the manually built that corresponds to a grouping of different SMAD proteins. An additional problem that made the comparison difficult was the fact that the researchers made different decisions concerning their map and model building. For instance, the receptor tyrosine kinase (RTK) component in the MAPK map represents several different receptors (e.g. EGFR, FGFR, VEGFR, etc.) while in the model they use explicitly the different receptors.

**Table 2.** Comparison of CaSQ-inferred Boolean models with manually built models (MM)

Map name	Map size	SBGN use	MM		CaSQ-inferred model BCC		Common nodes (%)
			Size	Graph reduction (%)	Size	Graph reduction (%)	
Mast cell	125	No	47	62	73	42	64
MAPK	232	No	53	77	181	22	79

The two models used for CaSQ’s benchmarking are medium-sized models (47–53 nodes). CaSQ models are twofold to fourfold bigger because they are inferred automatically from the corresponding maps (Table 2).

The CaSQ-inferred model for mast cell activation comprises 73 nodes while the manually built, 47 nodes. The authors of the manually built extracted information from the molecular map, but they also used proteomic data from bone marrow mononuclear cells (BMMCs) reported in Bounab *et al.* (2013) that focused on the SLP-76 protein and its partners. Node comparison revealed that 30 of these nodes are shared between the CaSQ inferred and the manually built models (Supplementary Table S1).

#### 3.3.2 *In silico* simulations and dynamic analysis

Next, we simulated CaSQ-inferred models to see if they were capable of capturing the system’s dynamics even though they were not identical with their manually built counterparts.

**Fig. 7.**
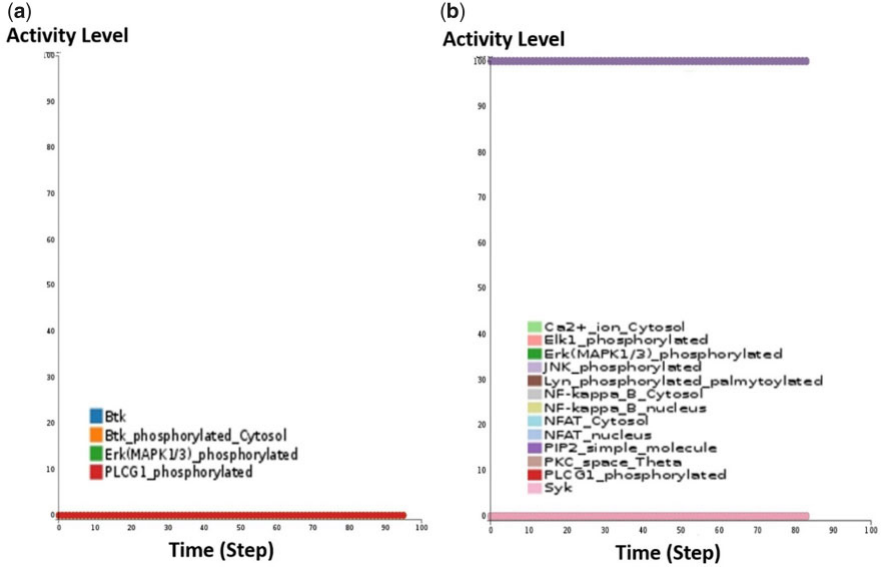
(**a**) Screenshot of simulations for Btk knockout of the CaSQ-derived mast cell activation model using Cell Collective. When Btk is set to zero, Erk and PLCG1 are not expressed. (**b**) Screenshot of simulations for Syk knockout of the CaSQ-derived mast cell activation model using Cell Collective. When Syk is set to zero, Erk, JNK, NFAT, NFkB, Ca2+, PKC, Elk1, PLCG1 are not expressed

*3.3.2.1 Comparison of the CaSQ-inferred model and the manually built model for mast cell activation*. One important difference, besides size and logical formulae, is also the fact that the mast cell activation model contained one multivariate variable while CaSQ-inferred models are strictly Boolean. Despite the differences, CaSQ mast cell model was able to reproduce the Btk (Fig. 7a) and Syk (Fig. 7b) knockout experiments described in the publication (Niarakis *et al.*, 2014).

In Figure 7, we see simulation examples of the CaSQ-inferred model for mast cell activation in Cell Collective.

In the case of Btk knockout, a decrease in cytokine release and degranulation, as well as a decrease of PLCG1and ERK levels have been observed (Kajita *et al.*, 2010; Setoguchi *et al.*, 1998). The simulation of Btk knockout using Cell Collective platform resulted in PLCG1 and ERK set to zero, a result that is directly comparable with the simulation described in Niarakis *et al.* (2014) (Fig. 7a).

In Syk knockout experiments, cytokine release and degranulation are both abolished (Gilfillan and Tkaczyk, 2006). We performed an *in silico* simulation of Syk knockout, with Lyn and PIP2 present at the initial state in Cell Collective as described in Niarakis *et al.* (2014) (Fig. 7b). In this condition, the CaSQ-inferred model reaches a state where ERK, JNK, Elk-1, NF-kB, NFAT, PKC, PLCG1, Ca2+ are all set to zero, in agreement with the simulation described in Niarakis *et al.* (2014).

*3.3.2.2 Logical steady-state analysis for the mast cell activation models*. We computed all the stable states of both the CaSQ-inferred model and the manually built one for mast cell activation using bioLQM java toolkit included in GINsim (http://colomoto.org/biolqm/). We obtained 18 stable states for the manually built model (Supplementary Fig. S4) and 524.288 for the CaSQ-inferred one. The difference in the number of stable states lies in the fact that the automatically inferred model is a close representation of the system as described in a molecular map and thus significantly bigger in size, including especially a much higher number of inputs. The manual counterpart is smaller in size and also of reduced complexity as several inputs are grouped and thus, the computation of stable states leads to considerably fewer solutions.

As shown in Supplementary Table S1, 30 components can be matched together between these two models. We then projected the identified stable states on these 30 components, which reduced the lists to nine stable states for the manually built model and 43.392 for the CaSQ-inferred one. Indeed, some of the original stable states only differ in the unmatched components and are thus projected on the same state. We found that three of the nine stable states of the manually built model are precisely reproduced in the CaSQ-inferred model. If we accept a single difference between the states, we can recover four additional stable states, whereas the last two stable states can be recovered with two differences (Supplementary Table S3).

*3.3.2.3 Comparison of the CaSQ-inferred model and the manually built model for MAPK*. Concerning the MAPK manually built model, the authors produced a model that did not follow strictly the corresponding map (the model contained several merged inputs and merged outputs).

**Fig. 8.**
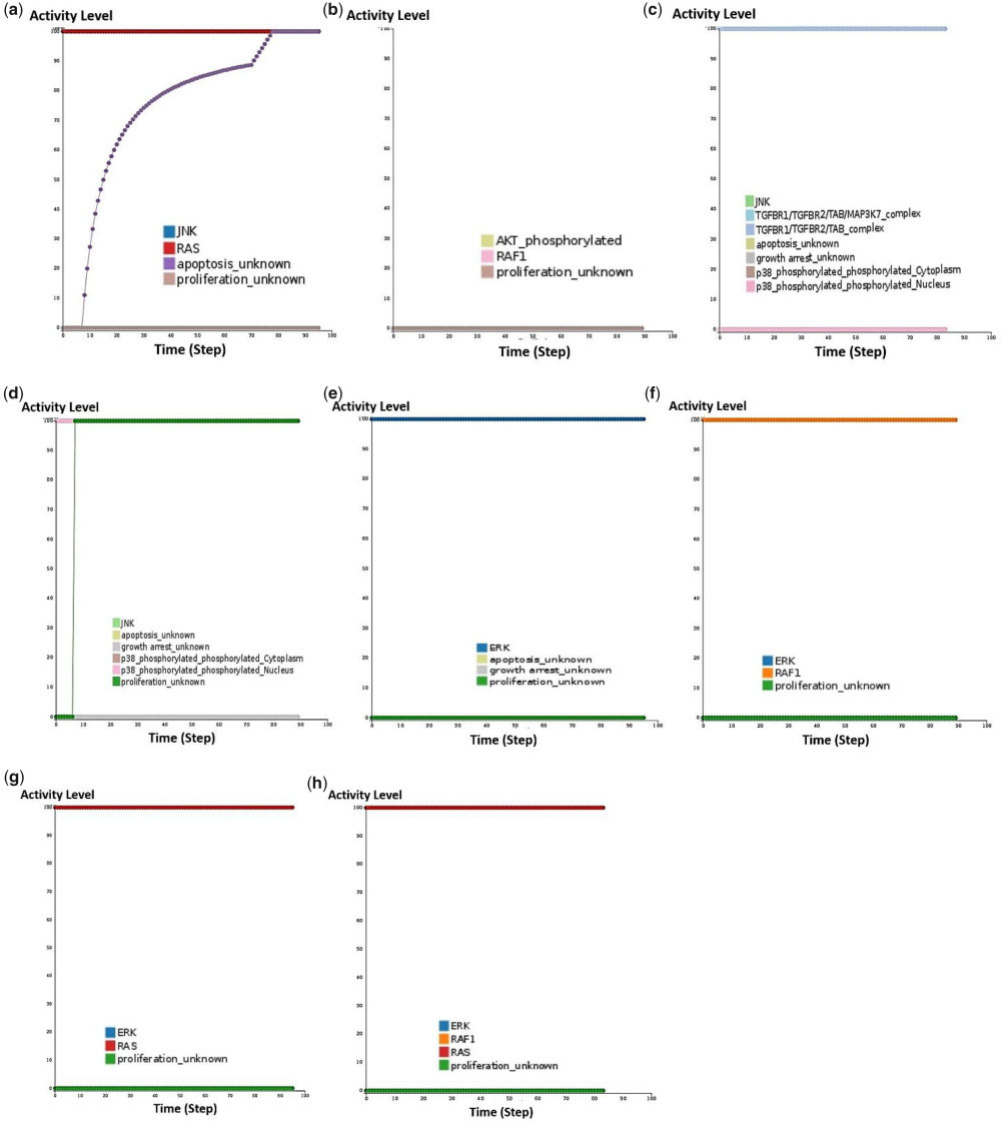
Simulations of the CaSQ-inferred model using the modelling platform Cell Collective. The CaSQ-inferred model for MAPK was able to reproduce known biological scenarios, either completely or partially. The results of the *in silico* simulations for the three first biological conditions described in Table 3 showed perfect agreement with the results of manually built model, as depicted in **a**, **b** and **c**. For conditions described in scenarios 4 and 5 of Table 3, the CaSQ-inferred model could partially reproduce the attended behaviour (**d** and **e**) while simulation results for scenario 6, were inconsistent with the literature and the results of the manually built model (**f**, **g** and **h**)

As stated above, the RTK component in the map represents several different receptors like EGFR, FGFR and VEGFR that the researchers decided to include in the model explicitly. Besides, to cope with simulations of their model, they used the model reduction option in GINsim (Grieco *et al.*, 2013) to produce different smaller sub-versions of the original model, each dedicated to a subset of simulations. In Table 3, we have regrouped biological scenarios modelled successfully with the MAPK manual model and the corresponding behaviour of the CaSQ counterpart. For the simulations of the CaSQ model, we used the platform Cell Collective as before (Fig. 8).

**Table 3.** Biological data and corresponding behaviours of the manually built and the CaSQ-inferred models for MAPK

Biological data	Manually built MAPK model	CaSQ-inferred MAPK model	Agreement
1.JNK might reduce RAS-dependent tumour formation by inhibiting proliferation and promoting apoptosis (Kennedy and Davis, 2003)	*When JNK is always ON and RAS is always ON then proliferation is OFF and apoptosis is ON*	*When JNK is always ON and RAS is always ON then proliferation is OFF and apoptosis is ON (Fig. 8a)*	Yes
2.HSP90 inhibitor disrupts EGFR, RAF and AKT leading to successful cancer treatment (Sharp and Workman, 2006)	*Concomitant RAF, EGFR, AKT deletions block proliferation*	***There is no EGFR present in the model*** *, RAF and AKT deletions lead to proliferation being OFF (Fig. 8b)*	Yes
3.P38 and JNK play important roles in stress responses such as cell cycle arrest and apoptosis (Kyriakis and Avruch, 2001; Takekawa *et al.*, 2011)	*When p38/JNK are OFF (KOs) and TGB and DNA damage are ON then there is no growth arrest or apoptosis*	***There is no DNA damage present in the model*** *, p38/JNK constitutively OFF and TGF stimuli ON, then Growth arrest is OFF and Apoptosis is OFF (Fig. 8c)*	Yes
4.P38 and JNK, especially in the absence of mitogenic stimuli, have been shown to induce apoptotic cell death (Kyriakis and Avruch, 2001; Takekawa *et al.*, 2011)	*When P38/JNK are constitutively ON then Growth arrest is ON, Apoptosis is ON and proliferation is OFF*	*When p38/ JNK are constitutively ON then* ***Growth arrest is OFF****, Apoptosis is ON and* ***proliferation ON****(Fig. 8d)*	Partial
5.ERK increases transcription of the cyclin genes and facilitates the formation of active Cyk/CDK complexes, leading to cell proliferation (Schramek, 2002)	*When ERK is always ON then Apoptosis and Growth arrest are OFF, and proliferation is ON*	*When ERK is constitutively ON then Apoptosis and Growth arrest are OFF, and* ***proliferation is OFF*** *(Fig. 8e)*	Partial
6.RAF or RAS overexpression can lead to constitutive activation of ERK (Dhillon *et al.*, 2007)	*When either RAS or RAF are constitutively active then ERK is ON and proliferation is ON*	*When either RAF or RAS or both of them are constitutively active, then* ***ERK is OFF*** *and proliferation is****OFF*** *(Fig. 8f–h)*	No

These reduced versions of the original MAPK model (52 components) ranged from 16 to 18 components. The CaSQ-inferred model for MAPK is inferred directly from the MAPK map and is thus significantly bigger in size and different in structure. However, comparison of the model’s behaviour regarding its efficacy in capturing the systems dynamics, showed that the CaSQ model, was able to reproduce partially or completely known biological scenarios.

The size of the CaSQ-inferred MAPK model (181 nodes) made the calculation of stable states a non-realistic endeavour. Moreover, the fact that the manually built counterpart had to undergo multiple reductions for the dynamic analysis, would not have made the comparison straightforward.

## 4 Discussion

Building large-scale dynamic models can be tedious and time-consuming work that requires not only the construction of the regulatory graph but also the writing and tuning of the logical formulae. CaSQ is a tool aiming to ease the construction of large-scale Boolean models, taking advantage of the similarities shared between molecular interaction maps and dynamic models. First of all, the molecular maps are process description representations that can be well annotated, providing a critical source of knowledge. The maps also contain information about the interactions, catalyzes, activations and inhibitions of the network, essential for the building of a computational model. In the framework proposed, we utilize systems biology standards for model construction (SBML-*qual*), so that CaSQ tool can be interoperable with other tools and modelling software.

An attempt to produce automatically large-scale models (kinetic and logical) has been made with the Path2Models (Büchel *et al.*, 2013) where researchers proposed a pipeline for the automatic generation of models using KEGG pathways as a resource. For metabolic pathways, they produced SBML files which they complemented where possible with kinetic data from respective databases, while for non-metabolic pathways, they produced SBML-*qual* files that could serve as scaffolds for logical models. These scaffolds do not contain logical rules, only topological relationships and interaction signs. In our pipeline, that requires only one tool, CaSQ, we start from detailed, mechanistic, process description diagrams and we produce fully executable large-scale logical models, with logical formulae for all components.

The methodology described in SQUAD (Mendoza and Xenarios, 2006) is complementary to what we propose and can be used in some parts of the obtained logical model if more quantitative evaluation is deemed necessary. For the inference of the logical formulae, we based our assumptions on topology and semantics of the molecular maps. More precisely, we decided to approach the conversion process using mostly OR gates over AND, so a target is on if one of the reactions producing it is on, a reaction is on if all reactants are on, all inhibitors are off and one of the catalysts is on. The idea behind this assumption is that very rarely we have exact information about the need for the presence of two or more activators for one target. Even if synergy is defined, very often a relative activation can happen even by the presence of one activator. Moreover, the number of events for which we do have such information is significantly lower than the uncertain ones and tuning the rules by hand should be a quick process.

The graph transformation rules that we use share some similarities with the rules used in http://pd2af.org, yet there exist significant differences: first, we do not address oligomerization as a specific case; instead, we chose to have a generic simplification for all complexes. On the contrary, we propose specific rules for receptors, as many of our use-cases have a signalling part which requires domain-specific rules. Concerning translocation, PD2AF does not make any simplification, whereas in our method, we have added a specific transport rule, as in the maps we treated we often encountered the case where an inactive form of a species is moving to another compartment and then becoming active (e.g. transcription factors). Ignoring the inactive version in the model did appear to correspond to what was done manually by the modellers in most of the cases studied.

Regarding activation and inhibition rules of PD2AF, our rules often agree except that we never extract the ‘hidden inhibition’ (or its converse): if there is an inhibition in the map, there will be an inhibition in the model, if there is an activation in the map, there is an activation in the model. While we understand the idea behind the PD2AF reasoning for this rule, the fact that it results in deleting the products of some reactions is in contrast with the reasoning behind CaSQ, which only deletes inputs. This is linked to the fact that an ‘inactive’ product can be a meaningful output of the map/model.

Finally, the most common catalytic reaction rule of PD2AF is different from our choice on several accounts. First, it uses a single state transition for all products of the reaction, which is not in the SBGN-AF standard. Furthermore, this single transition with multiple outputs makes it impossible to obtain specific logical rules for each of the outputs. In contrast, our methodology will duplicate the effect of reactants, activators and inhibitors for all products, i.e. create as many copies of the transition as there are products, and then combine this transition with all other transitions on each of those products. Moreover, the case of several activators/inhibitors is not covered by PD2AF, whereas we made a specific choice on how to combine them in a logical rule (AND’ing the reactants, OR’ing the activators and AND’ing the NEGation of all inhibitors). Finally, the most significant contrast to PD2AF, as already stated above, is that our resulting model is executable since it has inferred logical rules for each node.

Manually built models that are based on corresponding molecular maps are usually small to medium size because simulating a large-scale Boolean model remains challenging, even if the model is parameter-free. This means that the modeller is obliged to prioritize and choose nodes over others in order to create abstractions that can be subsequently analyzed. With the use of CaSQ, as demonstrated in this study, we can now obtain large-scale Boolean models that can be executed using popular modelling software that can import SBML-*qual* files. However, challenges associated with the analysis of large-scale Boolean models exist, and are active topics of efforts in the field. For coping with size and complexity one can perform reductions and create different versions of the original model [as demonstrated in Grieco *et al.* (2013)].

In this work, for comparing the tool’s performance and accuracy, we compared the common nodes between the CaSQ inferred and the manually built models, their ability to reproduce biological scenarios performing simulations, and finally, we performed a comparison of stable states, where possible. One problem we encountered when searching for common nodes was that the automatic comparison was not sufficient as a human modeller may choose different naming (e.g. merge two or more components). The automated comparison gave us an idea about the identical names and formulae, but a manual inspection was also compulsory as it revealed many cases where the corresponding nodes were present in both models, under slightly different naming. We also performed simulations to see if the CaSQ-inferred models could reproduce some of the dynamics of the original system. The next step was to perform logical steady-state analysis. For this purpose, we used GINsim, powerful software for logical modelling. The goal was to see if within the stable states of the CaSQ-inferred model, we could retrieve the stable states of the published manually built model.

We should note that CaSQ infers preliminary Boolean rules, so the modeller still needs to fine-tune the model and find the best logical rules to reproduce data accurately. Bekkar *et al.* (2018) show that logical models with added human curation perform better than models where rules are extracted automatically from a given topology. As demonstrated in the results, the CaSQ tool produces models that are largely in agreement with the model a human modeller would build, accelerating the time of model construction impressively.

This work was also a motivation for community work, as it addressed issues of model reusability, use of Systems Biology standard formats and interoperability between different tools that have complementary functionalities. As demonstrated, our method is scalable, and the large-scale SBML-*qual* models produced by CaSQ can be imported in Cell Collective and retain layout and annotations. However, the current import to GINsim requires a process that removes annotations and references before the analysis. Moreover, this process provides a solution for name display as GINsim displays species IDs that in our case make the model unreadable. The proper handling and reuse of annotations between different software tools could benefit from further interoperability work. The goal is to propose a seamless pipeline for producing executable Boolean models starting from molecular interaction maps which can be analyzed in depth using various tools for computational modelling. CaSQ tool can play the role of a bridge bringing together two distinct communities, curators and modellers to produce interoperable, annotated models of better quality, accuracy and reusability.

## 5 Conclusion—future prospects

CaSQ is a new tool for automated inference of Boolean models from CellDesigner molecular interaction maps. The rules defined for the translation have proven to be efficient to account for various biological scenarios, such as complex formation, protein activation, gene expression and transcription factor translocation. The obtained ‘raw’ models, with preliminary Boolean rules are able to reproduce complex behaviours and capture some of the systems dynamics. CaSQ can handle molecular maps varying significantly in terms of size, complexity, level of annotations and use of SBGN standards, with short run times. Finally, the obtained Boolean models retain the hierarchical layout of the map and its references in a standard format, SBML-*qual*, assuring model reusability and interoperability. The next step would be to use for downstream analysis of the CaSQ-inferred models, methods of probabilistic model checking to verify the correctness of our translation rules and the models’ sensitivity to their change (Abou-Jaoudé *et al.*, 2014; Bartocci and Lió, 2016; Traynard *et al.*, 2016). CaSQ-inferred models are compatible with tools like PRISM, a stochastic model checker (Kwiatkowska *et al.*, 2011) or MaBoSS, a software for simulating continuous/discrete time Markov processes, applied on a Boolean network (Stoll *et al.*, 2017). Performing in depth dynamical analysis of large-scale Boolean models and developing appropriate methodologies remain key challenges in the field of computational Systems Biology.

## Supplementary Material

btaa484_supplementary_data
