# Supplementary material for: Automated inference of Boolean models from molecular interaction maps using CaSQ
Source: Bioinformatics. 2020 May 13;36(16):4473–82. doi: 10.1093/bioinformatics/btaa484 (PMC7575051; doi:10.1093/bioinformatics/btaa484)
Supplement: btaa484_supplementary_data [file btaa484_supplementary_data.zip › Captions.docx]

Supplementary material

File 1: Executable files for CaSQ derived mast cell activation models (with default and BCC options

File 2: Executable files for CaSQ derived MAPK models (with default and BCC options)

File 3: Executable files for cholocystokinin models (with default and BCC options)

File 4: Executable files for Alzheimer’s models (with default and BCC options)

File 5: RA apoptosis executable modules (with default and BCC options)
