# Supplementary material for: Automated inference of Boolean models from molecular interaction maps using CaSQ
Source: Bioinformatics. 2020 May 13;36(16):4473–82. doi: 10.1093/bioinformatics/btaa484 (PMC7575051; doi:10.1093/bioinformatics/btaa484)
Supplement: btaa484_supplementary_data [file btaa484_supplementary_data.zip › Supplementary material_Figures_Tables.docx]

**Supplementary material**

**Figures and Tables**

***
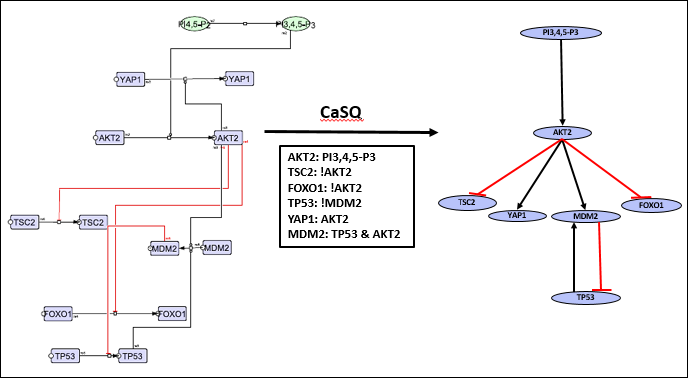
***

**Figure S1:** A complex part of the RA map translated into an activity flow (AF) like diagram with preliminary logical rules.

**
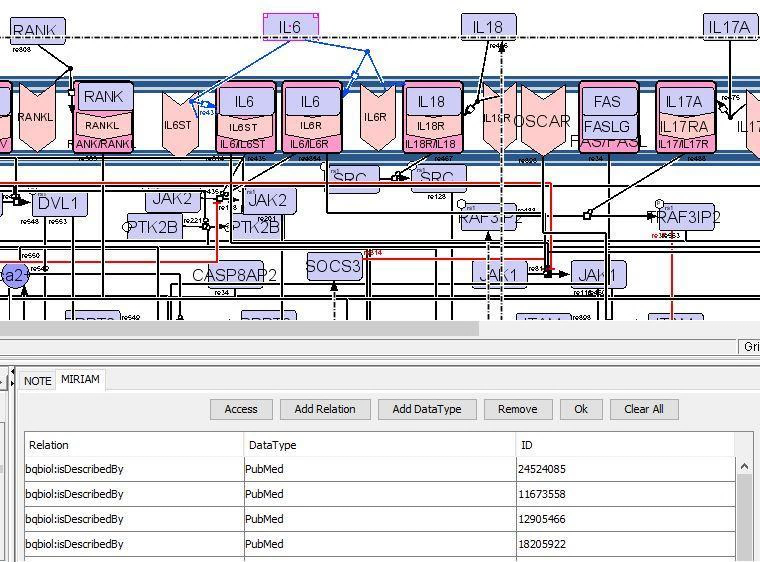
**

**Figure S2.** Snapshot of the RA map built with the software CellDesigner. The annotations for every node are stored in the MIRIAM section, using the bqbiol:isDescribedBy tag. Here we see the MIRIAM annotations for the node IL6.


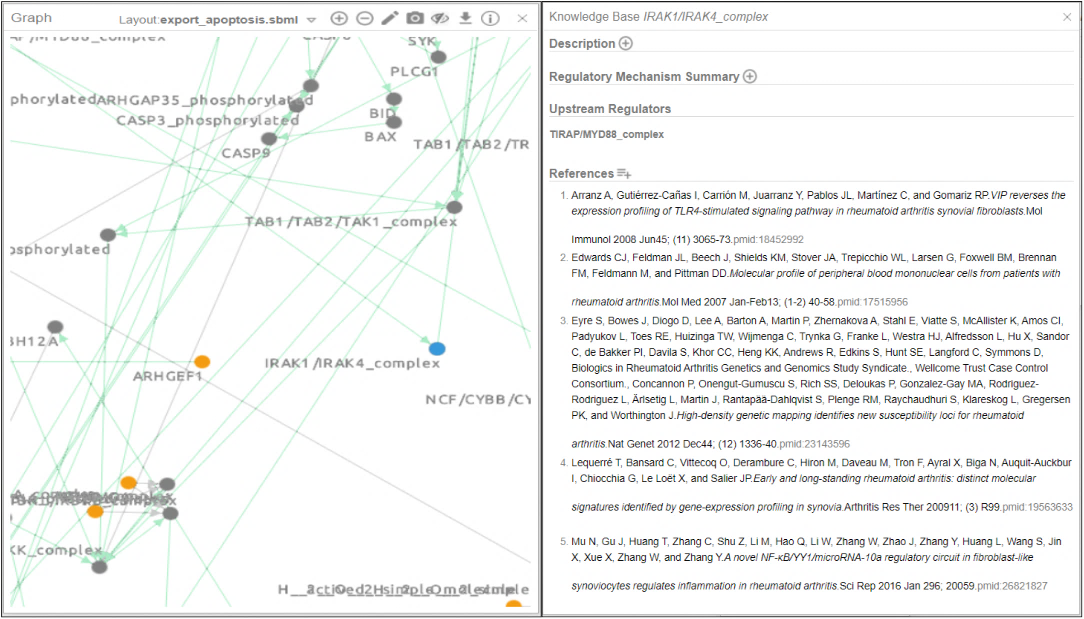


**Figure S3.** View of the CaSQ inferred models using the modelling platform Cell Collective. The CaSQ tool produces annotated Boolean models, including bibliographical references stored in MIRIAM. References stored in the MIRIAM section of the xml file of the molecular maps built with the software CellDesigner can be retrieved and visualized in the Cell Collective modelling platform. The original map layout is also conserved facilitating simulations. In the left panel, the node that corresponds to the IRAK1/IRAK4 complex is selected (node in blue color) while on the right panel the corresponding references are displayed.


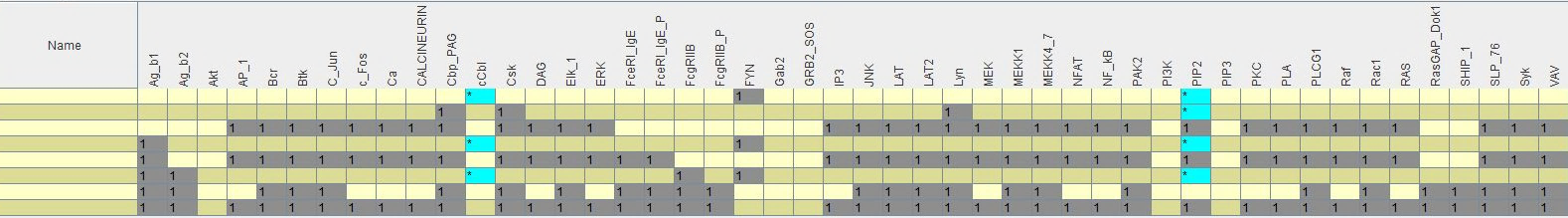


**Figure S4.** Screenshot of the stable states table for the manually built model for mast cell activation.

**Table S1**: Shared nodes between CaSQ and manually built model for mast cell activation.

| **Manual model** | **CaSQ produced model** |
| --- | --- |
| Bcr | Bcr |
| Btk | Btk, Btk_phosphorylated_Cytosol |
| C_Jun | c-JUN_phosphorylated_nucleus |
| c_Fos | Fos_phosphorylated |
| Ca | Ca2+_ion_Cytosol |
| Cbp_PAG | Cbp/PAG_palmytoylated |
| cCbl | cCbl |
| DAG | DAG_simple_molecule |
| Elk_1 | Elk1_phosphorylated |
| ERK | Erk(MAPK1/3)_phosphorylated |
| FYN | Fyn_phosphorylated_palmytoylated, Fyn_phosphorylated_phosphorylated_palmytoylated |
| Gab2 | Gab2 |
| IP3 | IP3_simple_molecule_Cytosol |
| JNK | JNK_phosphorylated |
| LAT | LAT1_phosphorylated_phosphorylated_phosphorylated_phosphorylated_palmytoylated |
| LAT2 | LAT2_phosphorylated_palmytoylated |
| Lyn | Lyn_phosphorylated_palmytoylated |
| MEKK1 | MEKK1(MAP3K1)_phosphorylated |
| NFAT | NFAT_Cytosol, NFAT_nucleus |
| NF_kB | NF-kappa_B_Cytosol, NF-kappa_B_nucleus |
| PIP2 | PIP2_simple_molecule |
| PKC | PKC_space_Theta |
| PLCG1 | PLCG1_phosphorylated |
| Raf | RAF1 |
| Rac1 | Rac1 |
| RAS | H-RAS |
| RasGAP_Dok1 | Dok1/RasGAP_complex |
| SHIP_1 | SHIP-1_phosphorylated |
| SLP_76 | SLP-76 |
| Syk | Syk_Cytosol |

**Table S2: Shared nodes between CaSQ and manually built model for MAPK.**

| **Manual model** | **CaSQ produced model** |
| --- | --- |
| AKT | AKT_phosphorylated |
| Apoptosis | apoptosis_unknown |
| ATF2 | ATF2_phosphorylated |
| ATM | ATM |
| BCL2 | BCL2, BCL2_gene |
| CREB | CREB1_phosphorylated |
| DUSP1 | DUSP1, DUSP1_don't care, DUSP1_gene |
| ELK1 | ELK1_phosphorylated |
| ERK | ERK, ERK_phosphorylated_phosphorylated_Cytoplasm, ERK_phosphorylated_phosphorylated_Nucleus, ERK_phosphorylated_phosphorylated_Mitochondria |
| FOS | FOS, FOS_phosphorylated, FOS_gene |
| FOXO3 | FOXO3_phosphorylated |
| FRS2 | FRS2_phosphorylated |
| GAB1 | GAB1 |
| GADD45 | GADD45_gene |
| GRB2 | GRB2 |
| Growth_Arrest | growth arrest_unknown |
| JNK | JNK, JNK_phosphorylated_phosphorylated_Cytoplasm, JNK_phosphorylated_phosphorylated_Nucleus |
| JUN | JUN_phosphorylated |
| MAP3K1_3 | MAP3K1, MAP3K2_3 |
| MAX | MAX_phosphorylated |
| MDM2 | MDM2, MDM2_gene |
| MEK1_2 | MEK, MEK_phosphorylated_phosphorylated |
| MSK | MSK_phosphorylated |
| MYC | MYC, MYC_gene |
| p14 | p14 |
| p21 | P21, p21_phosphorylated |
| p38 | p38_phosphorylated_phosphorylated_Cytoplasm, p38_phosphorylated_phosphorylated_Nucleus |
| p53 | TP53, TP53_phosphorylated |
| p70 | p70_phosphorylated |
| PDK1 | PDK1_unknown |
| PI3K | PI3K_phosphorylated |
| PKC | PKC |
| PPP2CA | PPP2CA_unknown |
| Proliferation | proliferation_unknown |
| PTEN | PTEN, PTEN_gene |
| RAF | RAF1, RAF1_phosphorylated_Cytoplasm, RAF1_phosphorylated_Mitochondria |
| RAS | RAS |
| RSK | RSK_phosphorylated |
| SMAD | Smad2, Smad3, Smad4, Smad2/Smad4_complex, Smad3/Smad4_complex |
| SOS | SOS |
| SPRY | SPRY_phosphorylated |
| TAOK | TAOK_phosphorylated |

**Table S3: Logical steady-state analysis obtained with GINsim, for manually built and CaSQ inferred mast cell activation models. The nine stable states of the manually built model are given in this table (SS1 to SS9). The bottom lines show which of these stable states are correctly recovered in the CaSQ inferred model, or have 1 or 2 mismatches.**

| Component | SS1 | SS2 | SS3 | SS4 | SS5 | SS6 | SS7 | SS8 | SS9 |
| --- | --- | --- | --- | --- | --- | --- | --- | --- | --- |
| Bcr | 0 | 0 | 0 | 0 | 0 | 0 | 1 | 1 | 1 |
| Btk | 0 | 0 | 0 | 0 | 0 | 0 | 1 | 1 | 1 |
| Ca | 0 | 0 | 0 | 0 | 0 | 0 | 1 | 1 | 1 |
| cCb1 | 0 | 0 | 0 | 0 | 0 | 0 | 0 | 1 | 1 |
| Elk1 | 0 | 0 | 0 | 0 | 0 | 0 | 0 | 1 | 1 |
| ERK | 0 | 0 | 0 | 0 | 1 | 1 | 1 | 1 | 1 |
| Gab2 | 0 | 1 | 0 | 1 | 0 | 0 | 0 | 0 | 0 |
| JNK | 0 | 0 | 0 | 0 | 0 | 0 | 0 | 1 | 1 |
| LAT | 0 | 0 | 0 | 0 | 0 | 0 | 1 | 1 | 1 |
| LAT2 | 0 | 0 | 0 | 0 | 0 | 0 | 0 | 1 | 1 |
| Lyn | 1 | 1 | 1 | 1 | 0 | 0 | 0 | 0 | 0 |
| NFAT | 0 | 0 | 0 | 0 | 0 | 0 | 0 | 0 | 0 |
| NFkB | 0 | 0 | 0 | 0 | 0 | 0 | 0 | 1 | 1 |
| PIP2 | 0 | 0 | 0 | 0 | 0 | 0 | 1 | 1 | 1 |
| PKC | 0 | 0 | 0 | 0 | 0 | 0 | 1 | 1 | 1 |
| PLCG1 | 0 | 0 | 0 | 0 | 0 | 0 | 1 | 1 | 1 |
| Rac1 | 0 | 0 | 0 | 0 | 1 | 1 | 1 | 1 | 1 |
| RAS | 0 | 0 | 0 | 0 | 0 | 0 | 1 | 1 | 1 |
| RasGAP-Dok1 | 0 | 0 | 0 | 0 | 0 | 0 | 0 | 1 | 1 |
| SHIP1 | 0 | 0 | 0 | 0 | 0 | 0 | 0 | 1 | 1 |
| SLP76 | 0 | 0 | 1 | 1 | 0 | 1 | 0 | 1 | 1 |
| Syk | 0 | 0 | 0 | 0 | 0 | 0 | 0 | 1 | 1 |
| C_Jun | 0 | 0 | 0 | 0 | 0 | 0 | 1 | 1 | 1 |
| c_Fos | 0 | 0 | 0 | 0 | 0 | 0 | 0 | 1 | 1 |
| Cbp_PAG | 0 | 0 | 0 | 0 | 0 | 0 | 1 | 1 | 1 |
| DAG | 0 | 0 | 0 | 0 | 0 | 0 | 0 | 1 | 1 |
| FYN | 0 | 0 | 0 | 0 | 0 | 0 | 1 | 0 | 1 |
| IP3 | 0 | 0 | 0 | 0 | 0 | 0 | 1 | 0 | 1 |
| MEKK1 | 0 | 0 | 0 | 0 | 0 | 0 | 1 | 1 | 1 |
| Raf | 0 | 0 | 0 | 0 | 0 | 0 | 1 | 1 | 1 |
| Exact match |  | x |  | x |  |  | x |  |  |
| One difference | x |  | x |  |  |  |  | x | x |
| Two differences |  |  |  |  | x | x |  |  |  |
